# Plan for Quarantine for Cholera

**Published:** 1866-05

**Authors:** 


*Plan for Quarantine for Cholera.*—We have received a plan for quarantine for cholera, originally addressed to the *Quebec Morning Chronicle*, by W. Marsden, M. D. It is based on the writer's study of the disease during five distinct epidemics, within the last thirty-four years. The basis of the plan is "absolute non-intercourse, for a short period, with persons from abroad suspected of being infected, and a thorough disinfection of personal effects." The paper contains a ground-plan of a quarantine-station, with a minute description, together with a strict code of rules for its management. The limit of detention for healthy persons, arriving in infected vessels, is ten days; and they are to be kept quite isolated from the sick.—*Boston Medical and Surgical Journal.*

